# Developing an Amplification Refractory Mutation System-Quantitative Reverse Transcription-PCR Assay for Rapid and Sensitive Screening of SARS-CoV-2 Variants of Concern

**DOI:** 10.1128/spectrum.01438-21

**Published:** 2022-01-05

**Authors:** Dongyan Xiong, Xiaoxu Zhang, Mengjuan Shi, Nuo Wang, Ping He, Zhuo Dong, Jie Zhong, Jing Luo, Yong Wang, Junping Yu, Hongping Wei

**Affiliations:** a CAS Key Laboratory of Special Pathogens and Biosafety, Center for Biosafety Mega-Science, Wuhan Institute of Virology, Chinese Academy of Sciences, Wuhan, China; b University of Chinese Academy of Sciences, Beijing, China; c Hubei International Travel Healthcare Center, Wuhan Customs Port Outpatient Department, Wuhan, China; University of Cincinnati

**Keywords:** SARS-CoV-2 variants of concern, conserved unique mutation, ARMS-RT-qPCR, rapid, sensitive

## Abstract

With the emergence and wide spread of severe acute respiratory syndrome coronavirus 2 (SARS-CoV-2) variants of concern (VOCs), such as the Delta variant (B.1.617.2 lineage and AY sublineage), it is important to track VOCs for sourcing of transmission. Currently, whole-genome sequencing is commonly used for detecting VOCs, but this is limited by the high costs of reagents and sophisticated sequencers. In this study, common mutations in the genomes of SARS-CoV-2 VOCs were identified by analyzing more than 1 million SARS-CoV-2 genomes from public data. Among them, mutations C1709A (a change of C to A at position 1709) and C56G, respectively, were found in more than 99% of the genomes of Alpha and Delta variants and were specific to them. Then, a method using the amplification refractory mutation system combined with quantitative reverse transcription-PCR (ARMS-RT-qPCR) based on the two mutations was developed for identifying both VOCs. The assay can detect as little as 1 copy/μL of the VOCs, and the results for identifying Alpha and Delta variants in clinical samples by the ARMS-RT-qPCR assay showed 100% agreement with the results using sequencing-based methods. The whole assay can be completed in 2.5 h using commercial fluorescent PCR instruments. Therefore, the ARMS-RT-qPCR assay could be used for screening the two highly concerning variants Alpha and Delta by normal PCR laboratories in airports and in hospitals and other health-related organizations. Additionally, based on the unique mutations identified by the genomic analysis, similar molecular assays can be developed for rapid identification of other VOCs.

**IMPORTANCE** The current stage of the pandemic, led by SARS-CoV-2 variants of concern (VOCs), underscores the necessity to develop a cost-effective and rapid molecular diagnosis assay to differentiate the VOCs. In this study, over 1 million SARS-CoV-2 genomic sequences of high quality from GISAID were analyzed and a network of the common mutations of the lineages was constructed. The conserved unique mutations specific for SARS-CoV-2 VOCs were found. Then, ARMS-RT-qPCR assays based on the two unique mutations of the Alpha and Delta variants were developed for the detection of the two VOCs. Application of the assay in clinical samples demonstrated that the current method is a convenient, cost-effective, and rapid way to screen the target SARS-CoV-2 VOCs.

## INTRODUCTION

The on-going pandemic caused by severe acute respiratory syndrome coronavirus 2 (SARS-CoV-2) has led to the deaths of millions of patients globally. Although SARS-CoV-2 vaccines are found to be effective in reducing the mortality significantly, the emergence of SARS-CoV-2 variants, especially those that are variants of concern (VOCs), may reduce the effectiveness of the vaccines and delay the end of the pandemic. Similar to other RNA viruses, the infectivity, transmissibility, and/or virulence of SARS-CoV-2 could be enhanced due to its high mutation rate during adaptive evolution within the host (1).

The first globally dominant mutation identified was D614G (a change of D to G at position 614) in the spike protein of SARS-CoV-2 (2–4). Some related studies revealed that the substitution D614G could enhance the viral transmission and replication in human cells (3, 5). After the emergence of the variant with the D614G mutation, it was reported that rapid transmission led by a SARS-CoV-2 variant was linked to infection among farmed minks (6). The outbreaks of these variants further exacerbated the severity of the COVID-19 pandemic, which promoted the need for tracking emerging variants by large-scale genome sequencing. In December 2020, British scientists first announced the identification of a novel SARS-CoV-2 variant via genome sequencing (7). This novel variant belonged to the lineage B.1.1.7 and had had a wide transmission in the world (8, 9). Subsequently, another SARS-CoV-2 variant, belonging to lineage B.1.351, was discovered in South Africa through genome sequencing in January 2021. It also spread quickly in 10 countries after it was first reported (10). Variants belonging to lineages B.1.1.7 and B.1.351 were found to have an N501Y amino acid substitution (8). Although both B.1.1.7 and B.1.351 strains showed reduced susceptibility to some neutralizing monoclonal antibodies, the strains from lineage B.1.351 had stronger resistance to neutralizing monoclonal antibodies than strains from lineage B.1.1.7. These B.1.351 strains were about 9.4-fold more refractory to neutralization by convalescent plasma, which was largely due to the E484K mutation in the spike protein (11). Another study revealed that, due to mutations K417N, E484K, and N501Y in the spike protein of B.1.351, convalescent and some vaccine sera might only offer limited protection against B.1.351 (12). More seriously, when the variants mentioned above had not been controlled effectively, a novel SARS-CoV-2 variant with high infection and mortality rates broke out in India in April 2021 (13). It rapidly evolved into three subtypes (B.1.617.1, B.1.617.2, and B.1.617.3) during its transmission (8). The variants of B.1.617.1 also showed reduced susceptibility to neutralization by sera from people vaccinated with the Moderna and Pfizer vaccines and from convalescent patients (14). In addition to the widespread SARS-CoV-2 variants described above, variants belonging to lineages like P.1, P.2, B.1.525, and B.1.526 were also found and of concern (8). Currently, from the latest VOC nomenclature recommended by WHO, the Alpha (B.1.1.7 and Q sublineages), Beta (B.1.351), Gamma (P.1), and Delta (B.1.617.2 and AY sublineages) belong to variants of concern, while others, such as Eta (B.1.525) and Iota (B.1.526), belong to variants under monitoring (VUMs) (https://www.who.int/publications/m/item/weekly-epidemiological-update-on-covid-19---16-november-2021). From the U.S. CDC’s variant clarification guidelines, the Delta variant was under VOC and the Alpha, Beta, and Gamma variants were under variants being monitored (VBMs) (https://www.cdc.gov/coronavirus/2019-ncov/variants/variant-info.html#Concern). In this study, we adopted the latest VOC nomenclature recommended by WHO. Overall, it is important to track these variants of concern (VOCs) to trace their transmission and study the protection efficacy of vaccines.

Next-generation sequencing (NGS) is the most accurate and widely used method for identification of SARS-CoV-2 VOCs since genome-wide mutations can be shown (15, 16). However, NGS represents a significant constraint for most resource-limited laboratories. Furthermore, it is technically difficult to get a complete virus genome when a sample contains low viral loads. Therefore, whole-genome sequencing is not a broadly accessible and cost-effective method to survey SARS-CoV-2 VOCs. The less-costly Sanger sequencing method is also used for VOC identification since most of the VOCs have various mutations in the gene of the spike protein (17), but it is not feasible for instant diagnosis. Another alternative method is to detect mutation sites based on PCR methods, such as amplification refractory mutation system quantitative reverse transcription-PCR (ARMS-RT-qPCR). ARMS-based PCR has been widely used to detect nucleotide point mutations associated with cancers (18, 19), antiviral drug resistance (20–22), Mycobacterium tuberculosis drug resistance (23), and SARS-CoV-2 variants (24). The PCR-based approach is easier to perform and more cost-effective than high-throughput sequencing. Since there are multiple mutations in variants, the current ARMS-PCR methods for screening SARS-CoV-2 variants depend on the combination of multiple hot spot mutations, such as D614G and R203K (24). But some of these hot spot mutations are presented in different variants and are not specific to a certain lineage. Moreover, costs for detection of multiple mutations are higher than for a single mutation.

In this study, the conserved and unique mutations of currently prevalent SARS-CoV-2 VOCs were analyzed through large-scale genomic analysis based on SARS-CoV-2 genomes from the public GISAID database in order to find mutations specific to a certain VOC. Then, an ARMS-RT-qPCR method was developed to detect the specific mutations for rapid and sensitive identification of SARS-CoV-2 VOCs. By detecting a single unique mutation site, the method provides a flexible and easy way to screen a target SARS-CoV-2 VOC.

## RESULTS

### Unique mutations in SARS-CoV-2 VOCs.

**FIG 1:**
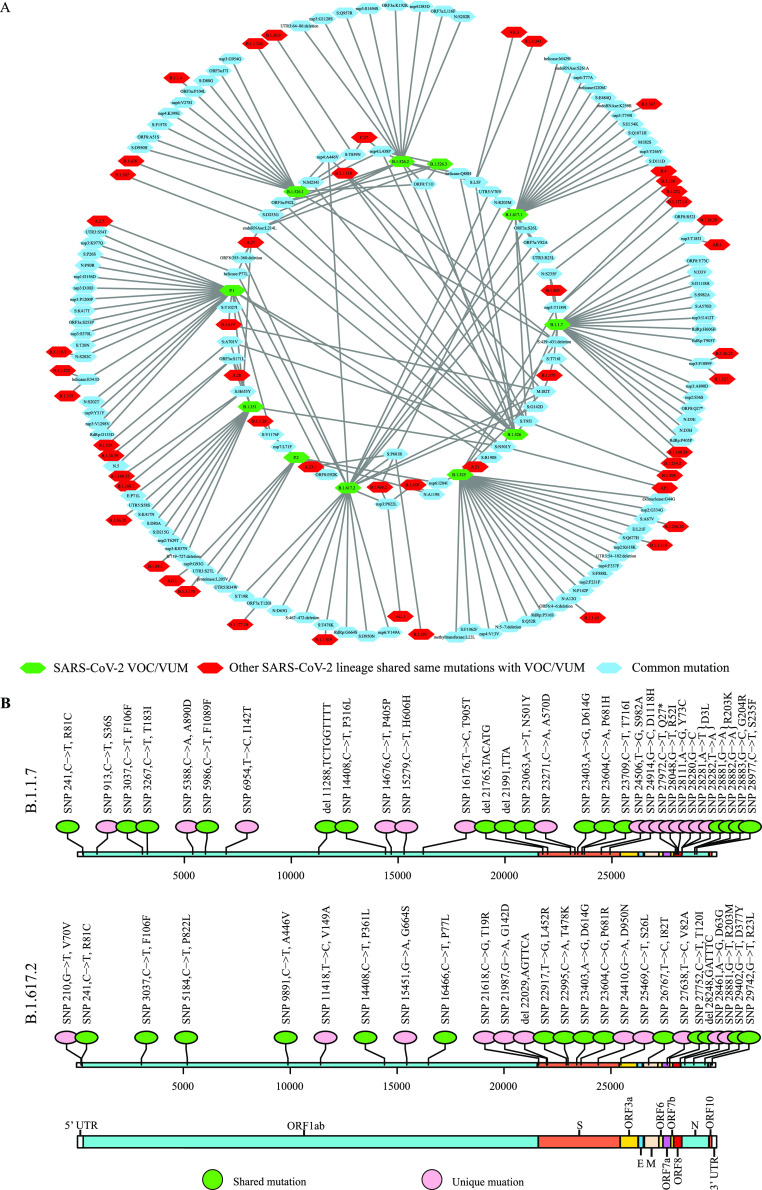
Identification of unique mutations of the SARS-CoV-2 variants of concern and variants under monitoring (VOCs and VUMs). (A) The relationship network between the common mutations of VOCs Alpha (B.1.1.7), Beta (B.1.351), Gamma (P.1), and Delta (B.1.617.2), VUMs Kappa (B.1.617.1), Eta (B.1.525), and Iota (B.1.526), and other lineages. Among these common mutations, each mutation connected with only one lineage was considered a unique mutation for the corresponding lineage and the others, linked to at least two different lineages, were regarded as shared mutations. Additionally, the mutations shared in more than 5 different lineages are not shown in the network. (B) The genome-wide common-mutation landscapes of the two rapidly and widely spread variants Alpha (B.1.1.7) and Delta (B.1.617.2).

SARS-CoV-2 genomes of high quality from December 2019 to three different time points were downloaded from GISAID and analyzed, and then the common mutations in each lineage of the Phylogenetic Assignment of Named Global Outbreak (Pango) system were obtained. The results showed that most of the lineages concerned had numerous mutations across their genomes, and the spike gene was found to contain common mutations for most VOCs (Fig. 1A, Table 1, and Table S1 in the supplemental material). Among the mutations, point mutations dominated (Fig. 1A and B). It is worth noting that the hot spot amino acid substitutions currently of interest, such as N501Y, E484K, and P681R, were not unique or specific mutations able to differentiate the VOCs. Therefore, none of these mutations could be used alone for identifying a certain lineage. However, as shown by the results in Tables 1 and 2, we found there were some highly conserved and unique mutations in the spike gene in the four rapidly and widely spread VOCs. One of the highly conserved mutations could be used alone for differentiating different VOCs. Because Alpha and Delta variants were dominant in the previous and current stages of the pandemic, these two variants were used as examples for developing the rapid detection method. The site mutations at the 1709th (position 23271 on the reference genome, A570D on the spike protein) and 56th (position 21681 on the reference genome, T19R on the spike protein) positions in the spike gene were chosen as the single targets for designing primer/probe sets for the ARMS-RT-qPCR to identify these two variants, respectively. In addition, we recently searched the two mutations A570D and T19R on the public website https://ngdc.cncb.ac.cn/ncov/lineage (25). The results clearly showed that these two unique mutations specific for the B.1.1.7 and B.1.617.2 lineages also covered the Q and AY sublineages of the Alpha and Delta variants, respectively. Thus, these two unique mutations, A570D and T19R, can be considered conserved and unique in all lineages of Alpha and Delta, respectively.

**TABLE 1:** The most conserved unique mutations in the spike gene in the four SARS-CoV-2 VOCs^*a*^

Lineage	Position	Nucleotide variation	Amino acid change	Frequency within lineage	No. of isolates
B.1.1.7 (Alpha)	23271	C→A	A570D	0.9983	605,322
	24506	T→G	S982A	0.9982	
	24914	G→C	D1118H	0.9980	

B.1.351 (Beta)	21801	A→C	D80A	0.9766	6,372
	22206	A→G	D215G	0.9254	
	22813	G→T	K417N	0.9232	
	22281–22289	Del:CTTTACTTG		0.9347	

B.1.617.2 (Delta)	21618	C→G	T19R	0.9990	33,411
	24410	G→A	D950N	0.9779	
	22029–22034	Del:AGTTCA		0.9544	

P.1 (Gamma)	21621	C→A	T20N	0.9927	18,524
	21638	C→T	P26S	0.9967	
	22812	A→C	K417T	0.9358	

a The analysis was based on more than 1,240,000 genomes from the GSAID database, and the lineage information from Pango lineages was used.

**TABLE 2:** Summary of several SARS-CoV-2 Pango lineages with conserved and unique mutations^*a*^

Mutation	% of isolates with mutation in lineage:							
	B.1.1.7	B.1.351	B.1.617.2	P.1	B.1.411	B.1.620	B.1.621	P.4
T19R	0	0	99.90	0	0	0	0	0
T20N	0	0	0	97.25	0	0	0	1.91
P26S	0	0	0	96.51	0	0.75	0	1.93
D80A	0	97.66	0	0	0	0	0	0
D215G	0	92.54	0	0	0	0	0	0
K417T		0	0	93.58	0	0	0	0
K417N	0	92.32	0	0	0	0	0	0
A570D	99.83	0	0	0	0	0	0	0
D950N	0	0	97.02	0	0	0	0.74	0
S982A	99.80	0	0	0	0.02	0	0	0
D1118H	99.74	0	0	0	0.02	0.02	0	0
Del:CTTTACTTG	91.34	0	0	0	0	1.90	0	0
Del:AGTTCA	0	0	95.44	0	0	0	0	0

a Over 1,240,000 genome sequences were analyzed to make this table. The proportion of 1 unique mutation in a specific lineage was calculated as follows: (number of isolates with the mutation from the specific lineage)/(total number of isolates from the specific lineage + total number of isolates from other lineages with the same mutation).

### ARMS-RT-qPCR assay for VOC screening.

SARS-CoV-2-positive samples (Table S2A) underwent RNA extraction and then were sent for NGS. After NGS analysis, seven nearly full-length SARS-CoV-2 genomes were obtained and found to be Alpha/Delta variants with the corresponding unique mutations described above (Fig. 1, Table 1, and Table S2C). Of the seven samples, two (SV001 and SV004) were identified as B.1.1.7 (Alpha) and B.1.617.2 (Delta) with mean sequencing depths of 21.91 and 918.47, respectively. These two samples with high viral loads were chosen for optimizing the ARMS-RT-qPCR assay. Additionally, the other samples with low viral loads that failed to get enough reads were sequenced by regular PCR-Sanger sequencing to find the specific mutations in the spike gene and for further validation (Table S2B).

**FIG 2:**
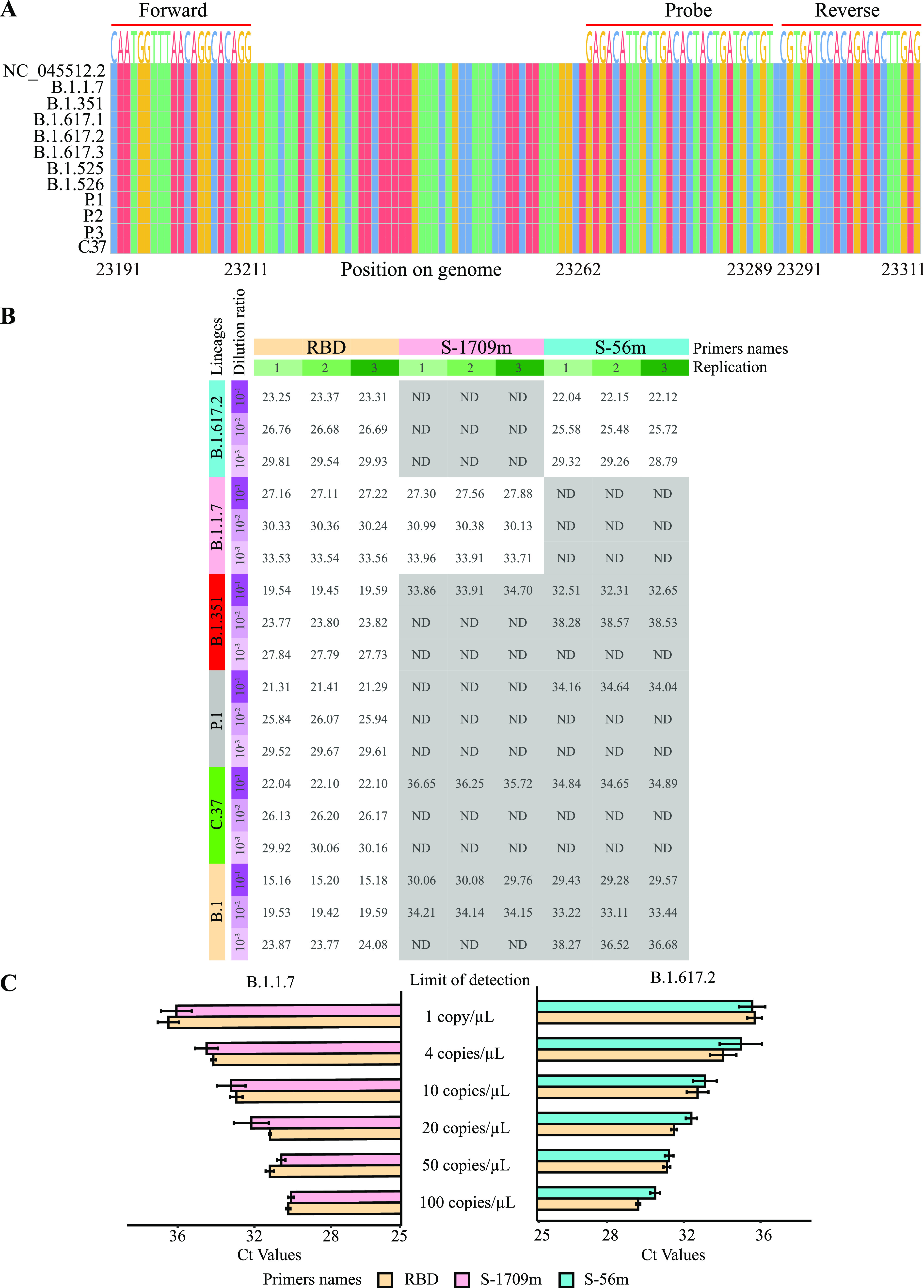
ARMS-RT-qPCR assay development. (A) Consistent comparison of the fragment amplified by the virus control targeting RBD in SARS-CoV-2 variants. (B) Validation of the two primer/probe sets for detecting the two VOCs (ND, not detectable). (C) Analytical sensitivity of the two primer/probe sets of the two VOCs to screen the corresponding VOCs. *C_T_*, cycle threshold. Gray shadows indicate that nucleic acid cannot be amplified effectively.

The unique mutations C1709A and C56G specific for the two VOCs (Alpha and Delta) were chosen for ARMS-RT-qPCR primer and probe design. After optimization, the best concentrations of primers and probe were found to be 0.4 μM and 0.1 μM, respectively. To better verify the accuracy of the mutation-specific primers and probes in distinguishing the target variants, plasmids of other variants Beta (B.1.351), Gamma (P.1), and Lambda (C.37) were synthesized. Together with the RNA extracted from the two samples (SV001 and SV004) and SARS-CoV-2 WIV04 (the B.1 lineage) (26), the optimized ARMS-RT-qPCR assays were used for identifying the two VOCs. The primers and probe for the virus control target a conserved fragment of the receptor-binding domain (RBD) shared by all of the SARS-CoV-2 variants (Fig. 2A), so that virus of the six lineages could be detected as shown in the columns headed “RBD” in Fig. 2B. At the same time, the two primer/probe sets targeting two VOCs could only amplify the samples containing the corresponding VOCs effectively, and not B.1 and other nontarget variants, as revealed by the cycle threshold (*C_T_*) values shown in Fig. 2B. These results clearly revealed that the primers and probes could effectively differentiate samples containing the target VOCs based on the Δ*C_T_* between the unique mutations of the VOCs and the conserved RBD fragment (the virus control). An average Δ*C_T_* of 13 was obtained when detecting nontarget lineages at high concentrations (low *C_T_* values, such as a *C_T_* of 15 and a *C_T_* of 19, as shown in Fig. 2B). When the virus concentration is low, there will be no amplification/*C_T_* value by the primer/probe set for the target VOC. In this case, in order to calculate the Δ*C_T_*, we used a *C_T_* of 45 for the calculation since the maximum number of cycles in the ARMS-RT-qPCR was 40. Furthermore, considering there would be some variations in *C_T_* values when detecting the same VOC using different primer/probe sets, a cutoff Δ*C_T_* of 5 was chosen, which was found to be robust enough to distinguish VOCs in different concentrations. Further testing of the dilution series of the two samples (SV001 and SV004) showed that an amount as low as 1 copy/μL of the target VOCs could be detected, which was about the same as for the virus control (Fig. 2C), indicating the high sensitivity of the two primer/probe sets.

### Performance of the ARMS-RT-qPCR-based assay for clinical samples.

**FIG 3:**
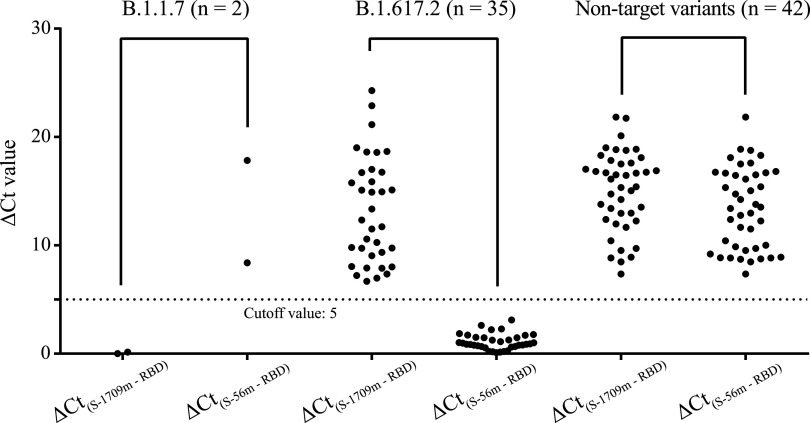
Determination of SARS-CoV-2 Alpha (B.1.1.7) and Delta (B.1.617.2) in clinical samples through the ARMS-RT-qPCR assay. *C_T_*, cycle threshold. Note that a *C_T_* value of 45 was used to calculate Δ*C_T_* when there was no amplification.

A total of 88 clinical swab samples were used for testing the performance of the ARMS-RT-qPCR developed here (Fig. 3). The 88 samples included 40 SARS-CoV-2-positive samples previously collected in January 2020 and sequenced by our laboratory (15) and 48 samples collected from suspected COVID-19 international travelers at the Wuhan international airport from May to July 2021. Among these specimens, 9 samples were found PCR negative for SARS-CoV-2 and 79 samples were found positive using commercial SARS-CoV-2 RT-PCR kits. All the SARS-CoV-2-positive samples could be detected by the virus control (targeting the conserved RBD fragment); the *C_T_* values are listed in Table S2A. Based on the cutoff value of 5 determined as described above and the Δ*C_T_* values of the PCR between the virus control and the two VOCs (primers S-1709m and S-56m, respectively), 2 of the 48 samples were identified as Alpha variant positive and 35 were identified as Delta variant positive. At the same time, all of the samples collected in 2020 were found not to contain the two VOCs tested. All these results were fully consistent with those of PCR-sequencing and NGS (Table S2B and C). The genomic results showed that the SARS-CoV-2 viruses in the samples collected in January 2020 were lineage A or B, which are the same as the early virus isolated in Wuhan when the epidemic started (26).

## DISCUSSION

During the worldwide spread of SARS-CoV-2 in the ongoing pandemic, at least four variants, Alpha (B.1.1.7 and Q sublineages), Beta (B.1.351), Delta (B.1.617.2 and AY sublineages), and Gamma (P.1) (8), have been called out for intense monitoring because they may be refractory to the protection provided by the current vaccines and have high transmissibility (11, 12). In the current study, we established a simple and rapid assay based on ARMS-RT-qPCR and targeting a single mutation each for the Alpha and Delta variants instead of using expensive whole-genome sequencing or detecting combined mutations to detect these variants (27, 28).

Based on over 1 million SARS-CoV-2 whole-genome sequences, the highly discriminant unique mutations of each lineage were identified by bioinformatics analysis (Table S1). The highly conserved mutations could be found across each VOC’s genome, especially in the spike gene (Fig. 1, Tables 1 and 2). According to the results from the bioinformatics analysis, it was possible to identify a single mutation that was unique to each lineage, covering over 99% of the genome sequences of each VOC. The results of determining the Alpha and Delta variants in clinical samples showed that targeting two mutations, at the 1709th and 56th positions of the spike gene, respectively, could identify the two target VOCs successfully within 2.5 h, which proved the rapidity of the developed ARMS-RT-qPCR assay. Because it is faster and less expensive than the current reported VOC detection methods based on the combination of hot mutations and NGS, we expect that this assay could be useful for tracking VOCs in the current epidemic, especially for resource-limited areas and laboratories. However, due to the good control of COVID-19 in China, only limited samples may be available for further validating the assay. Also due to the limited samples, detection of the other VOCs, such as Beta (B.1.351) and Gamma (P.1), was not included in the current study, but the unique mutations of these VOCs (Table 1) could be used for designing primers and probes to use the method to identify Beta, Gamma, or other SARS-CoV-2 variants. It should be noted that the PCR-based methods can only detect known mutations and the NGS-based approach is still the gold standard to determine novel SARS-CoV-2 variants, since NGS can report the unknown mutations from the samples.

In summary, an easy and sensitive ARMS-RT-qPCR approach was developed to reliably detect SARS-CoV-2 Alpha and Delta variants within 2.5 h by targeting a single unique mutation in each lineage. However, it will be best to check whether the unique mutation sites found are conserved for the variants with the spread of the pandemic by analyzing the continuously updated public genomic data from GISAID. Furthermore, the methods developed in this study provide a general way to determine other emerging variants.

## MATERIALS AND METHODS

### Analysis of public SARS-CoV-2 genomic data.

SARS-CoV-2 complete-genome data of high quality were downloaded from GISAID. The pairwise whole-genome alignment was performed by using the MAFFT software (version 7.471) (29). All mutations, including point mutations, deletions, and insertions, between the reference genome (accession number NC_045512.2) and each of the other genomes were determined by using an in-house script. In addition, the lineage information file for each public SARS-CoV-2 genome was downloaded from the Resource for Coronavirus 2019 database (https://ngdc.cncb.ac.cn/ncov/release_genome) (25). The lineage information declared by the database was calculated from the Pango lineages (Pangolin) database (https://cov-lineages.org/) (30). Combined with the lineage information, the mutations within each lineage were calculated by the in-house scripts. Briefly, the coverage of one mutation within a specific lineage was calculated as follows: (total number of genomes identified with this mutation from this lineage)/(total number of genomes from this lineage). Specifically, when determining the common mutations for one lineage, all mutations had to meet the following criteria: (i) there must be at least 100 genomes in one lineage, and (ii) only mutations presented in over 50% of the genomes within a lineage were considered common mutations in this lineage. After the analysis described above, files containing all common mutations in each lineage were archived. Further analysis to find the coverage of each mutation for each lineage was executed by using an in-house script. All of the code and in-house scripts can be found at https://github.com/MisgaXiong/Sripts_for_Paper/tree/master/SARS-CoV-2-mutation. The relationship network between common mutations of the SARS-CoV-2 VOCs and other lineages was visualized via Cytoscape software (version 3.7.2) (31). We analyzed all the public high-quality SARS-CoV-2 genomes at three time points: the time when Alpha (B.1.1.7) variants were dominant in the pandemic (6 March 2021), the time when Delta (B.1.617.2) variants caused concerns (14 May 2021), and the time when Delta variants were widely spread (13 July 2021). The numbers of genomes collected at the three time points for analysis were 366,529, 885,476, and 1,247,365, respectively.

### Sample collection and RNA extraction.

Pharyngeal swab samples used in the study were collected by Hubei International Travel Healthcare Center from international travelers for SARS-CoV-2 nucleic acid testing. These swabs were collected in viral transportation medium (VTM). Later, the samples were inactivated at 60°C for 30 min. RNA was extracted using the QIAamp viral RNA minikit (Qiagen, Hilden, Germany) according to the instructions of the manufacturer. The quality and quantity of the RNA were determined by Nanodrop (ND-2000; Thermo Fisher, Waltham, MA, USA). All samples were tested by RT-qPCR of a fragment in the sequence encoding the RBD in SARS-CoV-2 spike protein to screen for positive samples. The primers and probe used were as follows: forward primer, 5′-CAATGGTTTAACAGGCACAGG-3′; reverse primer, 5′-CTCAAGTGTCTGTGGATCACG-3′; and probe, 5′-FAM (6-carboxyfluorescein)-ACAGCATCAGTAGTGTCAGCAATGTCTC-BHQ1 (black hole quencher 1)-3′. The nucleic acids extracted from the positive samples were sent to the core facility of Wuhan Institute of Virology to perform next-generation sequencing.

### NGS.

The SARS-CoV-2-positive samples with high viral loads were analyzed by NGS. The NEBNext Ultra II directional RNA library prep kit (Illumina) was used for library construction. Sequencing was performed on the Illumina NextSeq 500 platform (Illumina, San Diego, CA, USA) utilizing the NextSeq 500/550 mid output kit version 2 (Illumina) producing 150-bp paired-end reads.

### High-throughput-data process.

Trimmomatic software (version 0.36) (32) was utilized to remove low-quality reads (<Q30) with the following options: LEADING, 20; TRAILING, 20; SLIDINGWINDOW, 4:20; MINLEN, 31. The resultant reads were aligned to the reference genome (accession number NC_045512.2) by using BWA software (version 0.7.17) (33) with the default options. The *de novo* assembly based on all mapped reads was performed by using megahit software (version 1.2.9) (34) with the default options. After that, the nearly full-length SARS-CoV-2 genome was obtained. The strain typing of the sequenced viruses was determined by Pango lineage (https://cov-lineages.org/) (30), and the mutations of these isolates were analyzed by the method described above.

### Primer and probe design.

**FIG 4:**
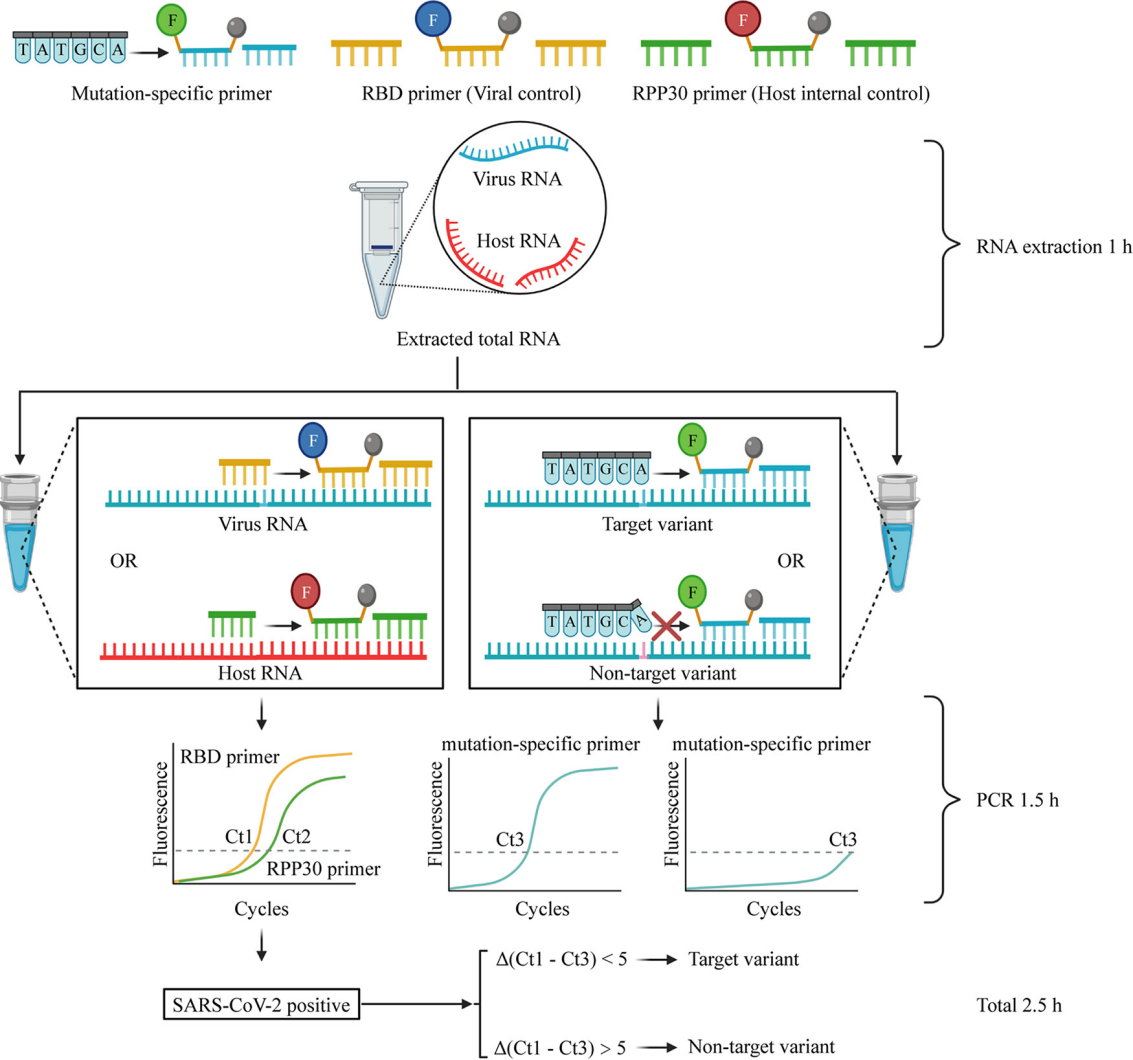
Scheme of the process of the ARMS-RT-qPCR assay for detecting SARS-CoV-2 variants.

Based on the unique mutations from the genomic analysis, ARMS-RT-qPCR primers and TaqMan probes for screening VOCs were designed using Beacon Designer (version 8.13; Premier Biosoft) and Primer Premier 5.0 software. Briefly, as shown in Fig. 4, one primer was designed with a base at the 3′ end matching the nucleic acid base of a VOC so that it would amplify the VOC fragment efficiently but not amplify other variants. The design of the probes and the reverse primers was the same as for ordinary qPCR. Two sets of primers and probe targeting two conserved and unique mutations were designed for the detection of Alpha and Delta variants, respectively. In detail, the primers and probes for ARMS-RT-qPCR are listed in Table 3 and the regular primers targeting the spike gene for validation of unique mutations are listed in Table 4. All the primers and probes used were synthesized at Sangon Biotech Co., Ltd. (Shanghai, China).

**TABLE 3:** Primer and probe sets for ARMS-RT-qPCR for the two VOCs

Variant	Nucleic acid mutation (amino acid change)	Primer	Direction	Sequence (5′–3′)^*a*^
Alpha	C1709A (A570D)	S-1709m	Forward	CAACAATTTGGCAGAGACATTAA
			Reverse	CCAAGTAGGAGTAAGTTGA
			Probe	5′-HEX-TCCACAGACACTTGAGATTCTTGACA-BHQ1-3′

Delta	C56G (T19R)	S-56m	Forward	GTCAGTGTGTTAATCTTAG
			Reverse	GAACCAAGTAACATTGGAA
			Probe	5′-ROX-ACATTCAACTCAGGACTTGTTCTTACC-BHQ2-3′

a HEX, 6-carboxy-2,4,4,5,7,7-hexachlorofluorescein; BHQ, black hole quencher; ROX, carboxy-X-rhodamine.

**TABLE 4:** Primers for the PCR-sequencing used to confirm the variants by Sanger sequencing

Primer	Amplicon size (bp)	Position on the gene	Direction	Sequence (5′–3′)
Spike-500-1	495	1–495	Forward	CTTCTTAGTAAAGGTAGACTTATAATTAGAG
			Reverse	CTTCAAGGTCCATAAGAAAAGGCTGAGAG
Spike-Special-2	365	2839–3203	Forward	ATAGTGCTATTGGCAAAATTCAAGACTC
			Reverse	CATGACAAATGGCAGGAGCAGTTGTGAAG

### ARMS-RT-qPCR assay for Alpha and Delta variant determination.

Two SARS-CoV-2-positive samples (SV001 and SV004) with *C_T_* values of 27.16 and 23.85, respectively, were chosen for ARMS-RT-qPCR assay development. The process of the ARMS-RT-qPCR assay for Alpha and Delta variant determination is illustrated in Fig. 4. Briefly, two duplex assays in two PCR tubes were performed to detect these two variants in clinical samples. Each duplex assay consisted of two primer/probe sets. The first duplex assay is the same as in our previous study (35). One primer/probe set (virus control) was used to amplify the conserved RBD gene fragment, and another set (internal control) was used to amplify the human RPP30 gene. In the second duplex assay, one primer/probe set was used to target C1709A for identifying the Alpha variant, and another set was used to target C56G for identifying the Delta variant. Reverse transcription-quantitative PCR (RT-qPCR) was performed using the HiScript II one step qRT-PCR probe kit (Vazyme Biotech Co., Ltd., Nanjing, China). The RT-qPCR assay mixture was composed of the primers and the probes with optimized concentrations, 10 μl 2× one step Q probe mix, 1 μl one step Q probe enzyme mix, 5 μl RNA template, and nuclease-free water to a final volume of 20 μl. All qPCR experiments were performed in a CFX96 Touch real-time PCR detection system (CFX96; Bio-Rad Laboratories, Hercules, CA, USA) with an initial reverse transcription step at 50°C for 15 min, denaturation at 95°C for 30 s, and then 40 cycles of denaturation at 95°C for 10 s and annealing at 57°C for 30 s with fluorescent signal acquisition. The determination of variants was based on the difference between the *C_T_* values targeting the unique mutations of the VOCs and the *C_T_* values targeting the conserved RBD fragment (the virus control) (Δ*C_T_*). If the Δ*C_T_* value was less than 5, the sample was regarded as the corresponding VOC. Otherwise, if the Δ*C_T_* value was larger than 5, (23) the sample was considered not to contain the corresponding VOC (nontarget VOC).

### Limit of detection determination.

Samples SV001 and SV004 were 2-fold serially diluted in phosphate-buffered saline (PBS) buffer and then used as the templates for the ARMS-RT-qPCR in triplicate after RNA extraction. The lowest concentrations that could be detected were the limits of detection for the two VOCs, and the lowest copy numbers were calculated based on the standard curve between the *C_T_* values and the copy numbers targeting RBD based on droplet digital PCR (ddPCR) assays performed on the Bio-Rad QX200 ddPCR system previously (36).

### Detection of SARS-CoV-2 variants in clinical samples.

A total of 88 clinical samples (pharyngeal swabs) were tested for the detection of Alpha and Delta variants using the ARMS-RT-qPCR method developed as described above. All the variants were verified by PCR-sequencing of the gene for the spike protein using the primers listed in Table 4, which covered multiple highly discriminant unique mutations of the SARS-CoV-2 VOCs. The multiple unique mutations revealed by the sequencing can confirm the VOCs.

### Data availability.

The sequenced genomics data of SARS-CoV-2 reported in this study have been deposited in the Genome Warehouse in the National Genomics Data Center (https://ngdc.cncb.ac.cn/gwh) (37), Beijing Institute of Genomics (China National Center for Bioinformation), Chinese Academy of Sciences. The BioProject accession number is PRJCA005985. The accession numbers of the submitted genomes are GWHBEBP00000000, GWHBEBW00000000, GWHBEBX00000000, GWHBEBY00000000, GWHBEBZ00000000, GWHBECA00000000, and GWHBECB00000000.
